# Arsenic abrogates the estrogen-signaling pathway in the rat uterus

**DOI:** 10.1186/1477-7827-8-80

**Published:** 2010-07-02

**Authors:** Aniruddha Chatterjee, Urmi Chatterji

**Affiliations:** 1 Department of Zoology, University of Calcutta, 35 Ballygunge Circular Road, Kolkata-700019, India

## Abstract

**Background:**

Arsenic, a major pollutant of water as well as soil, is a known endocrine disruptor, and shows adverse effects on the female reproductive physiology. However, the exact molecular events leading to reproductive dysfunctions as a result of arsenic exposure are yet to be ascertained. This report evaluates the effect and mode of action of chronic oral arsenic exposure on the uterine physiology of mature female albino rats.

**Methods:**

The effect of chronic oral exposure to arsenic at the dose of 4 microg/ml for 28 days was evaluated on adult female albino rats. Hematoxylin-eosin double staining method evaluated the changes in the histological architecture of the uterus. Circulating levels of gonadotropins and estradiol were assayed by enzyme-linked immunosorbent assay. Expression of the estrogen receptor and estrogen-induced genes was studied at the mRNA level by RT-PCR and at the protein level by immunohistochemistry and western blot analysis.

**Results:**

Sodium arsenite treatment decreased circulating levels of estradiol in a dose and time-dependent manner, along with decrease in the levels of both LH and FSH. Histological evaluation revealed degeneration of luminal epithelial cells and endometrial glands in response to arsenic treatment, along with reduction in thickness of the longitudinal muscle layer. Concomitantly, downregulation of estrogen receptor (ER alpha), the estrogen-responsive gene - vascular endothelial growth factor (VEGF), and G1 cell cycle proteins, cyclin D1 and CDK4, was also observed.

**Conclusion:**

Together, the results indicate that arsenic disrupted the circulating levels of gonadotropins and estradiol, led to degeneration of luminal epithelial, stromal and myometrial cells of the rat uterus and downregulated the downstream components of the estrogen signaling pathway. Since development and functional maintenance of the uterus is under the influence of estradiol, arsenic-induced structural degeneration may be attributed to the reduction in circulating estradiol levels. Downregulation of the estrogen receptor and estrogen-responsive genes in response to arsenic indicates a mechanism of suppression of female reproductive functions by an environmental toxicant that is contra-mechanistic to that of estrogen.

## Background

Arsenic is a naturally occurring metalloid with potent toxic and mutagenic effects [1]. It is present ubiquitously in the environment and is released from both natural and man-made sources [2]. Arsenic in drinking water is one of the topmost environmental threats worldwide, based on the potential exposure of people to arsenic and the numerous diseases with which it has been associated [3-6]. In Southeast Asian countries like India, Bangladesh and Taiwan, millions of people are threatened by arsenic poisoning, leading to several diseases and disorders, and even death [7]. The problem of arsenic poisoning is not only restricted to developing countries but developed nations like USA, Germany, China, Japan and Australia are also plagued by problems of arsenic contamination [8,9]. Interestingly, inorganic arsenic is found to be more potent than the organic form and trivalent compounds are found to be more toxic than pentavalent ones [10].

Chronic intake of arsenic is strongly associated with an increased risk of skin, lung, liver and other cancers, type 2 diabetes, cardiovascular diseases, neurological and cognitive defects, and reproductive and developmental problems [11-16]. According to World Health Organization, the permissible limit of arsenic in drinking water is 0.01 mg/l, which is equivalent to 10 ppb [9,15,17,18]. Recently, however, it has been reported that there is an increased risk of arsenic toxicity, even at the low and permissible dose of 10 ppb [9,15-17]. However, a large population all over the world is exposed to far higher levels of arsenic [19-23]. In certain areas in the Indian subcontinent, the maximum arsenic concentration in ground water was found to be around 3700 ppb [24] to 4700 ppb [18], leading to several physiological damages to human beings. Although arsenic is not a direct acting xenotoxin or mutagen, it may increase DNA damage or mutations indirectly by altering DNA repair, thus acting as a co-carcinogen or promoter of tumor growth [25].

Till date, there is very little information regarding the mechanism of arsenic action on the ovarian steroidogenic function and the female reproductive axis, particularly in wide areas of India and other countries, where the levels occurring in drinking water exceed the admissible limits of 10 ppb [20-22,24]. It is, however, known that women who are exposed to this level of arsenic often suffer from spontaneous abortion and stillbirth [26], and maternal exposure to arsenic also affects the health of newborn and promotes carcinoma incidences in them [27-29]. Although it has been hypothesized that the reproductive hazards may be due to disruption of the steroid hormone signaling pathway [30,31], the actual target of arsenic is probably a part of the mechanism which is used to regulate gene expression and not just the receptor itself [31]. The estrogen receptor (ER) is the most divergent of all steroid hormone receptors and may undergo divergent co-regulatory interactions and unique activation/de-activation steps. Previous studies have suggested that arsenic can interfere with ER functioning, although the exact mechanism remains to be ascertained [31]. Since arsenic acts as a potent environmental estrogen [32], it was explicable to study the arsenic-stimulated estrogen receptor signaling pathway, expression of estrogen responsive genes such as VEGF, and G1 cell cycle regulatory proteins CDK4 and cyclin D1, since these molecules are known to be primary responsive factors to estrogen administration in the rat uterine endometrium [33-35]. Thus, the objective of this study was to elucidate the role of arsenic as an endocrine disruptor and determine the molecular mechanism underlying arsenic action in the rat uterus.

## Methods

### Animals

Female Sprague-Dawley rats, aged 15-16 weeks and weighing 100-120 g, were collected from the breeding colony and maintained under controlled conditions (25 ± 2°C temp, 50 ± 15% RH and normal photoperiod 12 h dark and 12 h light) through out the experiment. The animals were given sterile food pellets and water *ad libitum *and allowed to acclimatize to the laboratory environment for 5 days prior to the commencement of the experiments. The Principles of Laboratory Animal Care (NIH Publication No 85-23, revised in 1985) as well as specific Indian Laws of Animal Protection (ILAP) were followed through out the experimental schedule.

### Drug treatments, selection of optimum dose and time and study of estrous cycle

Sodium arsenite (E-Merck, Germany) was used for study. All other chemicals were procured from Sigma Aldrich (USA). The rats were divided randomly into different groups, each containing 5 animals. Group I (control group) animals were fed 10-12 ml pure distilled water/animal/day, while other groups were fed the same volume of water containing sodium arsenite at different concentrations (0.4 μg/ml, 4 μg/ml, 40 μg/ml and 80 μg/ml) [27-29,36-38] and maintained for different time periods (7 days, 14 days, 28 days and 56 days) in order to determine the optimum dose and time of arsenic action. The optimum time and dose of arsenic action, as determined by circulating estradiol concentrations, were selected for all subsequent experiments. The dose of arsenic thus selected conforms to environmentally relevant concentrations. Vaginal smears were collected every morning from all of the animals before fresh treatment of arsenic. The smears were double stained with eosin-hematoxylin and examined microscopically.

### **Tissue and blood collection**

Animals were anesthetized by intraperitoneal injection of sodium barbital. Uteri were quickly removed from the experimental animals and washed in 0.9% (w/v) cold normal saline, pat dried and weighed in an electrical monopan balance (Lutron GM-300 P). Small representative tissue slices were processed for histological and immunohistochemical studies, RNA isolation and protein purification. Blood was collected from the heart and serum was isolated for ELISA.

### Assay of serum estradiol, LH and FSH

ELISA (DRG International ELISA Kit) was performed for estimating the circulating levels of estradiol, LH and FSH. For assay of serum estradiol, 25 μl each of standard, control and treated serum samples were added to respective wells coated with anti-estradiol antibody and incubated with 200 μl of enzyme conjugate for 2 hours at room temperature. Subsequently, 100 μl of substrate was added and incubated for 15 minutes at room temperature. Reactions were stopped using 50 μl of stop solution and the O.D. was measured at 450 nm. Each sample was run in triplicate [39]. For assay of serum FSH and LH, 25 μl of standard, control or treated serum samples were added to respective wells coated with anti-FSH and anti-LH antibodies and incubated with 100 μl of enzyme conjugate for 30 minutes at room temperature. Wells were washed with *aqua dest *and 100 μl of substrate was added to each well. After incubation for 10 minutes at room temperature, reactions were stopped using 50 μl of stop solution and the O.D. measured at 450 nm [38,39]. The intra-assay and inter-assay variations were found to be less than 9% and 10%, respectively. Limit of detection for estradiol was 3.6 pg/ml, for LH was 0.45 mIU/ml and for FSH was 0.28 mIU/ml.

### Histology and morphometric analysis

Uterine slices (selected randomly from the proximal, middle and distal regions of the uterus) from control and treated animals were fixed in bouins fluid. Graded dehydration of the tissue was done by 70 to 100% alcohol in subsequent steps and xylene was used as the clearing agent. For histological studies, 5 μ paraffin sections were stained by standard hematoxylin-eosin double staining procedure and observed under a microscope [31]. The stained sections were subjected to morphometric analysis using the eye piece scale (occulometer) and the stage micrometer. The stage and the eye piece scales were adjusted until there was a parallel point between the two scales. The number of the eye piece divisions and its corresponding stage measurements was noted. The occulometer fixed into the microscope was then focused through stained sections of the tissue to allow for measurement of the luminal diameter, height of luminal epithelial cells, size of endometrial glands and longitudinal muscle layer [40].

### Immunohistochemistry

Immunohistochemistry was performed according to a previously established protocol [26]. Briefly, sections were deparaffinized, hydrated, boiled in 0.1 M sodium citrate buffer (pH 6.0) for 10 minutes and treated with 3% (v/v) H_2_O_2 _in PBS (30 min) to block endogenous peroxidase activity. Non-specific staining was blocked by incubating the sections with bovine serum at room temperature. Sections were subsequently incubated with rabbit anti-ER IgG (sc-542, Santa Cruz Biotech, CA, 1:500) for 18 hours at 4°C and then with goat anti-rabbit HRP-conjugated secondary antibody (1:1000) for 1 hour at room temperature. Peroxidase was visualized using 3,3'-diaminobenzidine. The sections were counterstained with hematoxylin. Slides that served as negative controls were not incubated with the primary antibody [27].

### RNA extraction and semi-quantitative RT-PCR

Uterine tissues were collected from the arsenic-treated and untreated rats. 100 mg of tissue samples were frozen quickly in liquid nitrogen and total RNA was isolated using TRI reagent (SIGMA), dissolved in DEPC water and quantified by UV spectrophotometry. The RNA samples were subjected to DNase treatment prior to RT-PCR. Reverse transcription reaction was performed at 42°C with 5 μg of RNA in 5× reaction buffer (Fermentas, USA) containing 100 pmol random hexamer primer, 10 mM dNTP mixture (Fermentas, USA), 20 units of RNase inhibitor (Bioline, USA) and 200 units of RevertAid™ M-MuLV Reverse Transcriptase (Fermentas, USA). PCR was initiated using 2.5 μg cDNA, 10 mM dNTPs and 1 unit of Taq DNA Polymerase (Vivantis, USA). PCR amplifications were performed using the primers listed in Table 1[41-45]. PCR was carried out for 40 cycles using an annealing temperature of 58°C for ERα and 55°C for VEGF, CDK4 and cyclin D1. Samples were fractioned by 2% agarose gel electrophoresis and quantified using a BioRad Gel Documentation System.

**Table 1:** Sequences of the oligonucleotides used for semi-quantitative RT-PCR

Gene	Forward primer	Reverse primer	**Ref**.
ER α	GGAGACATGAGAGCTGCCAAC	CCAGCAGCATGTCGAAGATC	[41]
VEGF	GATCAAGTTCATGGACGTCT	GATCAAGTTCATGGACGTCT	[43]
Cyclin D1	CTGGCCATGAACTACCTGGA	GTCACACTTGATCACTCTGG	[45]
GAPDH	GACATCAAGGTGGTGAAGCAG	CACCCTGTTGCTGTAGCCATATTC	[42]
CDK4	TGGTGTCGGTGCCTATGGGA	GGTAGCTGTAGATTCTGGCT	[44]

### Western blot analysis

Tissue samples were lysed in ice-cold RIPA Buffer (150 mM NaCl, 50 mm Tris, 0.1% Triton X-100 and 0.1% SDS containing protease inhibitors [4-(-2-aminoethyl benzenesulphonyl fluoride), EDTA, leupeptin, aprotinin and bestatin, SIGMA]. The concentration of total protein was determined by Bradford assay and equal amount of proteins (30 μg) were fractioned by 8% SDS-PAGE. Proteins were electrically transferred to PVDF membranes and blocked for 2 hours at room temperature with 5% non-fat dry milk. Blots were subsequently incubated with ERα, VEGF and CDK4 antibodies raised in rabbit and cyclin D1 antibody raised in mouse (1:1000), for 18 hours at 4°C. Anti-β tubulin was used as a loading control. Blots were subsequently incubated with HRP-conjugated goat anti-rabbit and goat anti-mouse IgG secondary antibodies (1:2000), respectively, for 1 hour at 25°C. Immunoreactive proteins were detected by staining the membranes with 3,3'-diaminobenzidine in 50 mM Tris (pH-7) containing 0.2% H_2_O_2 _[46] and bands were quantified by a BioRad Gel Documentation System.

### Statistical analysis

Results of the experiments performed in triplicates were expressed as mean and standard error of mean of different groups, using a statistical software package (Graphpad). The differences between the mean values were evaluated by one-way ANOVA followed by multiple Students' t-tests. P-values less than 0.05 were considered statistically significant [47,48]. Densitometric analysis of the RT-PCR and western blot results were carried out using NIH Scion Image analysis to assess the fold-change in arsenic-treated rats as compared to the control animals.

## Results

### Effect of arsenic on food consumption, body weight and estrous cycle

It was observed that during the entire duration of the experiment, water intake and food consumption of the control and treated rats remained unchanged. In addition, the body weights of the treated animals were not significantly different from that of the control ones. However, after 28 days of arsenic treatment, a significant decrease in the wet weight of uterus was observed in comparison to the control group (Table 2). In addition, in the control group, regular estrous cycles of 4-5 days were noted whereas, in the arsenite-treated group, a constant diestrous phase was observed after 22 ± 2 days of arsenic treatment.

**Table 2:** Body weight and uterine wet weight in response to 4 μg/ml arsenic treatment for 28 days in adult female rats

Treatment	Body weight (in gm)	Uterine wet weight (in mg)
Control	132 ± 1.67	146 ± 2.31
4 μg/ml NaAsO_2_	131.6 ± 1.32	104.3 ± 1.94**

Individual weights of control and arsenic-treated rats were attained at the end of the experimental schedule. Each value represents mean ± SE, n = 5. (ANOVA followed by multiple Students' t-tests, ** p < 0.01, as compared with respective control).

### Dose and time-dependent effect of arsenic on serum estradiol levels

**Figure 1:**
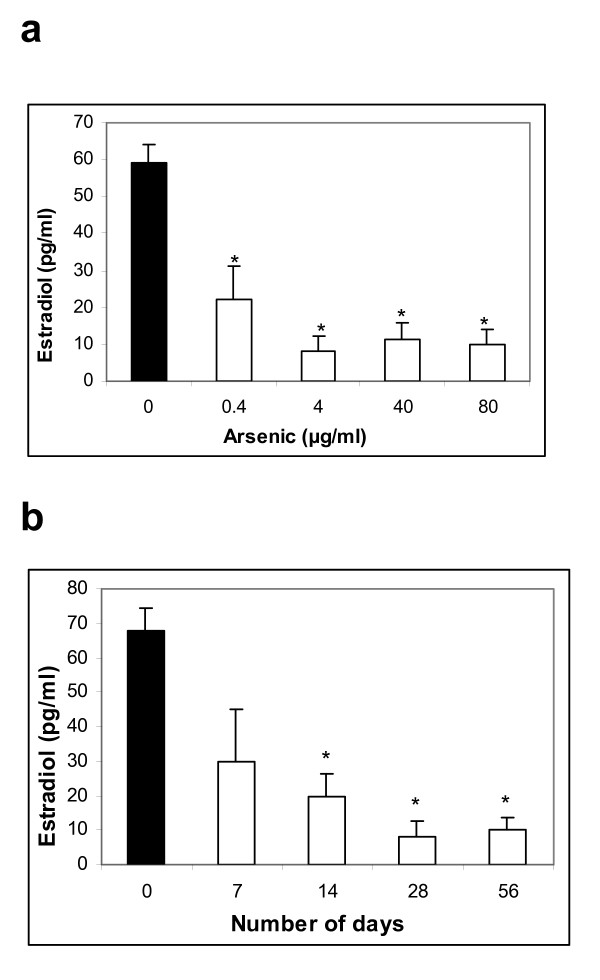
**Serum estradiol levels of untreated and arsenic treated female Sprague Dawley rats**. Rats were exposed to different doses of sodium meta-arsenite for different time periods, as mentioned in the *Methods *section. (a) Arsenic has a dose-dependent inhibitory effect on circulating estradiol levels. The dose at which maximum reduction is observed is 4 μg/ml, beyond which the level does not change significantly. Each value represented as mean ± SE, n = 3, p < 0.05. (b) The minimum time period required to initiate the maximum decrease in estradiol levels is 28 days. No further reduction is observed even when rats were exposed to arsenic for 56 days. Each value represented as mean ± SE, n = 3, p < 0.05.

In order to determine the effect of arsenic on serum estradiol levels in female Sprague-Dawley rats, the optimum dose responsible for the changes and the minimum time required to initiate maximum changes were resolved by ELISA of serum estradiol. Rats were treated with different doses of sodium arsenite and monitored at the end of 28 days. As depicted in Figure 1a, the serum estradiol levels reduced at a dose of 0.4 μg/ml and decreased by 4-fold in rats fed with arsenic-containing water at a dose of 4 μg/ml, as compared to control rats (p < 0.05). The level did not decrease further when the rats were fed arsenic-containing water at the dose of 40 μg/ml and 80 μg/ml. The minimum time required for maximum effect of arsenic was resolved to be 28 days, beyond which continuous treatment did not affect the serum estradiol levels up to a period of 56 days (Figure 1b). Hence, all subsequent experiments were performed with the optimum effective dose and time of 4 μg/ml (equivalent to 4 ppm) and 28 days, respectively.

### Effect of arsenic on serum concentration of LH and FSH

**Figure 2:**
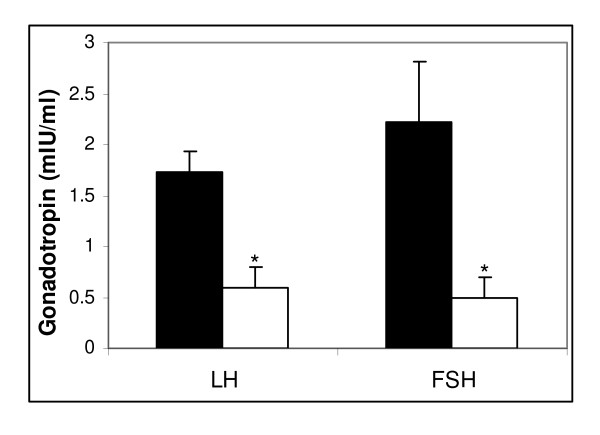
**Serum gonadotropin levels of untreated and arsenic-treated female rats**. Sprague-Dawley rats were treated with 4 μg/ml sodium meta-arsenite for 28 days, as described in the *Methods *section. The results indicate that arsenic has an inhibitory effect on serum levels of both LH and FSH (white bars), as compared to the control animals (black bars). Each value represented as mean ± SE, n = 3, p < 0.05.

LH and FSH are gonadotropins which are upstream components of estradiol signaling, and are required for the development and quantitative maintenance of normal reproductive cycle in pubertal rats. It was observed that chronic exposure of rats to 4 μg/ml sodium arsenite for 28 days significantly decreased the serum LH and FSH concentrations (Figure 2), as detected by ELISA. Synthesis and secretion of estradiol is under the control of these gonadotropins, hence rendering them important components of sex steroid regulation. Thus, the reduction of LH and FSH may therefore be responsible for the consequent reduction in estradiol levels, as seen above.

### Arsenic-induced histological changes of the uterus

**Figure 3:**
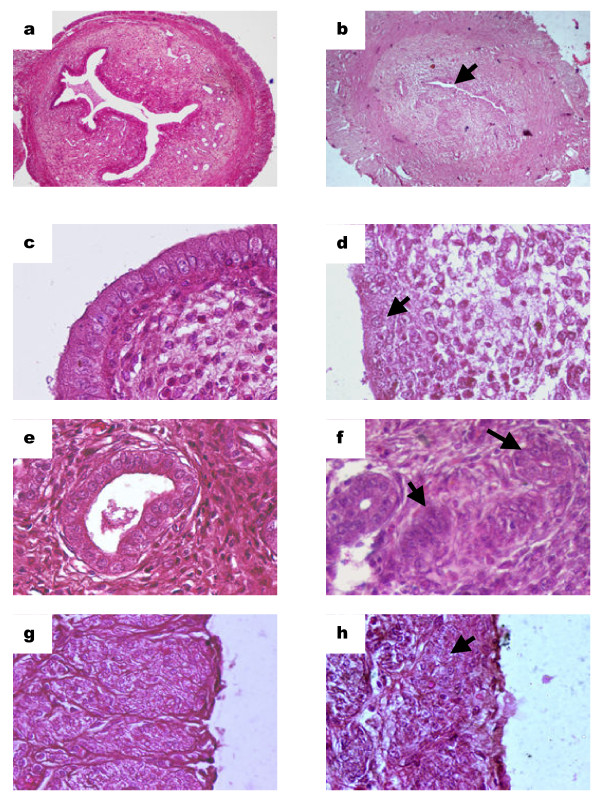
**Alterations in tissue architecture of arsenic-treated rat uterus**. Rats were treated with or without 4 μg/ml arsenic for 28 days. 3a, 3c, 3e and 3g: control sections; 3b, 3d, 3f and 3h: arsenic-treated sections. Uterine sections were subjected to double staining with hematoxylin and eosin and examined under the microscope. (3a, 3b) A significant decrease in width and invaginations of the arsenic-treated uterine lumen was observed in the treated sections (10× magnification) as compared to untreated one. (3c, 3d) Reduction in the height of luminal epithelial cells (as measured by occulometry) in the arsenic-treated sections was observed as compared to untreated one (40×). In addition, degeneration of the layer was also noted in the treated uterus. (3e, 3f) Arsenic treatment resulted in a significant reduction in the size of endometrial glands, with disappearance of the lumen and disorganization of the epithelial cells lining the glands. (3g, 3h) As compared to the control uterus, significant reduction in size and organization of the longitudinal muscle layer was observed in the arsenic-treated uterine sections.

Histological analysis of 4 μg/ml arsenic-treated rats showed significant alterations in the uterine morphology as compared to the untreated rats. Occulometric studies revealed rats that were exposed to arsenic showed a decrease in (i) the size and invaginations of the uterine luminal diameter, (ii) height of luminal epithelial cells, (iii) number and organization of endometrial glands, and (iv) the width of the myometrium (Table 3). The compact, tall, columnar epithelial cells lining the highly invaginated lumen of the untreated uterus (Figure 3a) were well defined as compared to the treated uterus (Figure 3b), with rounded nuclei located on a prominent basement membrane (Figure 3c). However, the height and organization of the epithelial cells were affected with arsenic treatment and revealed distortion of the cells with irregular-shaped nuclei, along with disappearance of a distinct basement membrane (Figure 3d). Additionally, the number and size of endometrial glands in the endometrial stroma were well defined in the control uterus (Figure 3e) and were significantly reduced in the treated animals (Figure 3f). Reduction in the thickness of the longitudinal muscles comprising the myometrium, as compared to the untreated uterus (Figure 3g), was also observed in the uterus of rats exposed to arsenic (Figure 3h).

**Table 3:** Luminal diameter, height of luminal epithelial cells, diameter of endometrial glands and width of longitudinal muscles in different experimental groups of adult female rats

Treatment	Luminal diameter (μm)^#^	Height of luminal epithelial cells (μm)^§^	Diameter of endometrial glands (μm)^§^	Width of longitudinal muscle layer (μm)^§^
Control	45.88 ± 1.87	18.44 ± 0.19	58.95 ± 3.04	75.89 ± 0.68
4 μg/ml NaAsO_2_	10.72 ± 0.51**	5.7 ± .04***	16.61 ± 0.44**	37.69 ± 1.6**

Histological analysis was carried out for each control and arsenic-treated rat, and the uterine parameters were measured by occulometry. Each value represents mean ± SE, n = 5. (ANOVA followed by multiple Students' t-tests, ** p < 0.01, *** p < 0.001, as compared with respective controls). ^# ^Magnification: 10×; ^§ ^magnification: 40×.

### Effect of arsenic on estrogen receptor expression

**Figure 4:**
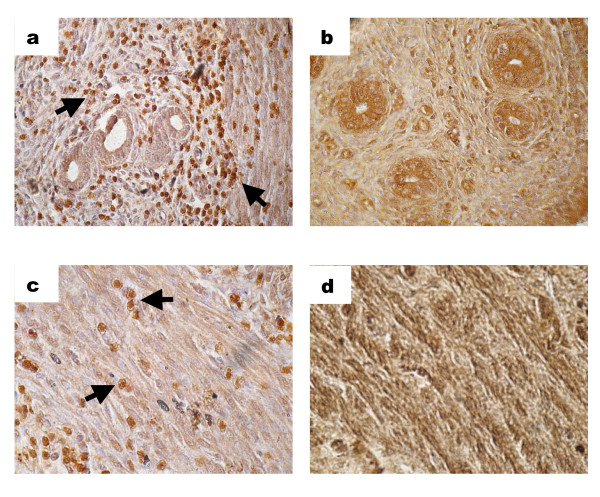
**Immunohistochemical localization of estrogen receptor in the rat uterine sections**. Rats were treated with arsenic for 28 days and tissues were stained for the presence of the estrogen receptor. Arsenic significantly downregulated the estrogen receptors in the endometrial stroma (4b) as compared to the untreated uterus (4a). In addition, the expression of estrogen receptors was also decreased in the longitudinal muscle layer (4d) in contrast to the expression in the muscle layer of the control uterus (4c).

**Figure 5:**
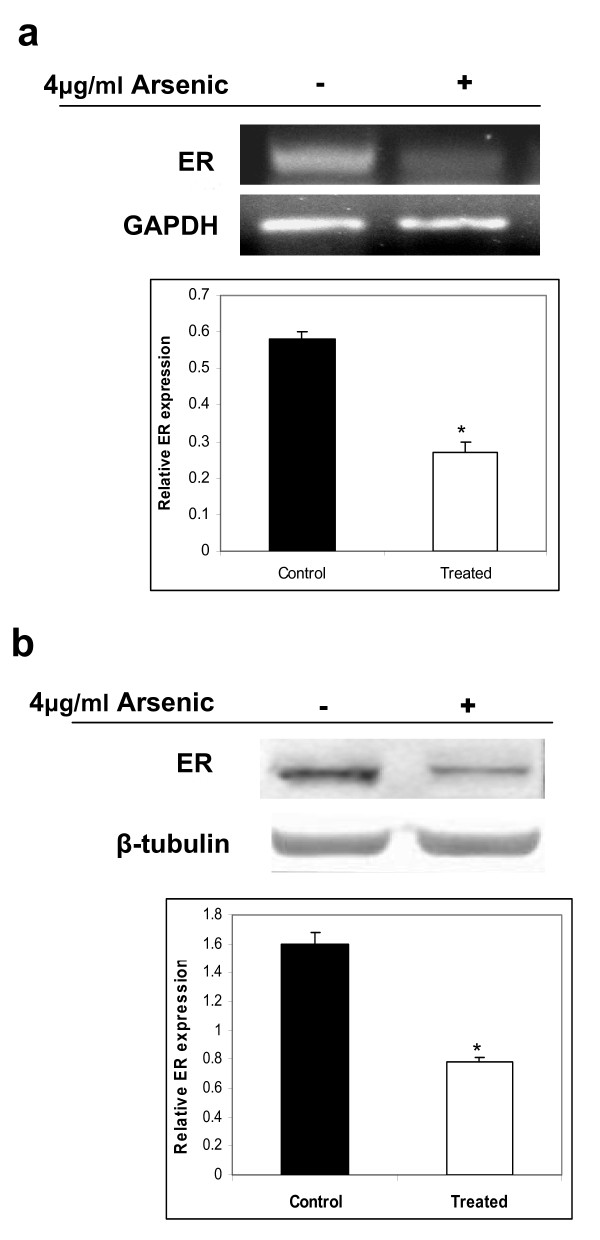
**Effect of arsenic on estrogen receptor expression at the mRNA transcript and protein levels**. Downregulation of the estrogen receptor in the arsenic-treated uterus was observed both at the mRNA transcript levels, as determined by RT-PCR (a), and at the protein levels, as determined by western blot analysis (b). The data shown is a representative of the experiments carried out in triplicate.

Immunohistochemical localization of estrogen receptors were detected by staining for the ERα proteins in the uteri of arsenic treated and untreated rats. Uterus from untreated rats showed presence of ERα (Figure 4a) whereas rats exposed to arsenic showed significant downregulation of ERα in the uterine endometrium (Figure 4b). Similar results were observed in the muscle layer of the control (Figure 4c) and the arsenic-treated uterus (Figure 4d). In order to further validate the effect of arsenic on the expression of estrogen receptors in the rat uterus, the expression was monitored both at the RNA transcript levels and at the protein levels. Semi-quantitative RT-PCR confirmed that the level of ERα was downregulated as a result of arsenic treatment as compared to the control uterus (Figure 5a). Western blot analyses further confirmed that concentration of the estrogen receptor protein declined by almost 2-fold as a result of exposure to arsenic (Figure 5b).

### Effect of arsenic on vascular endothelial growth factor

**Figure 6:**
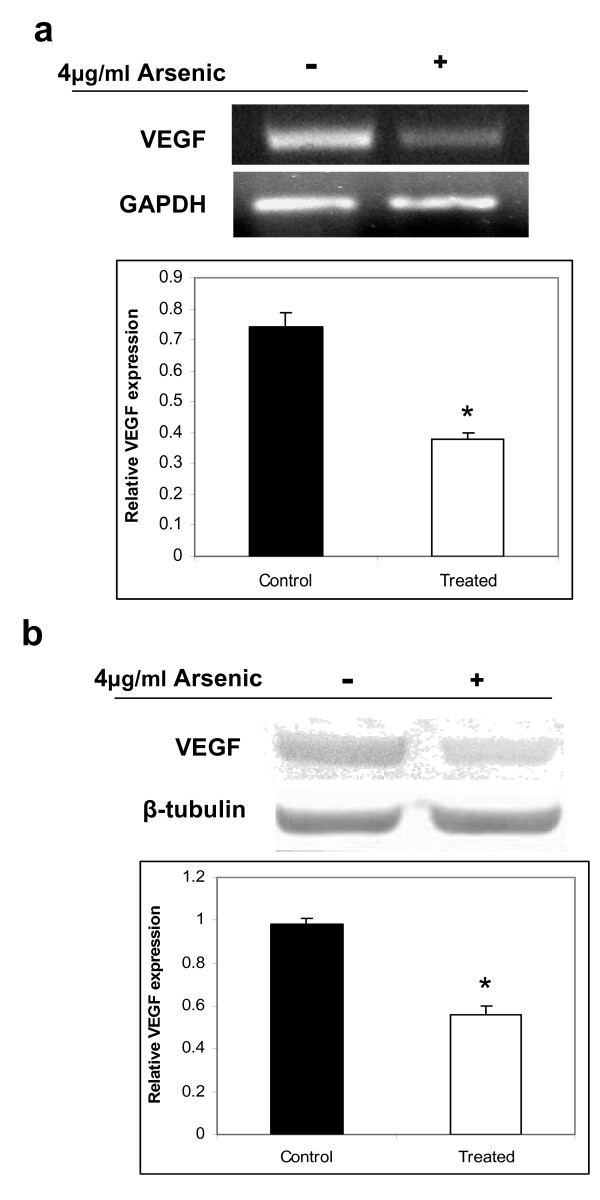
**Arsenic downregulated VEGF expression in the rat uterus**. Expression of VEGF, an estrogen-responsive gene, was observed to be downregulated both at the transcriptional (a) and translational levels (b), as determined by semi-quantitative RT-PCR and western blot analysis, respectively, in the uterus of arsenic-treated rats. The data shown is a representative of three independent experiments.

Since arsenic is a potent environmental estrogen and responsible for downregulation of the estrogen receptor, we investigated the effects of arsenic on the estrogen signaling pathway. Concomitantly, we selected vascular endothelial growth factor (VEGF) as the estrogen-responsive gene, since it is known to respond primarily to estrogen administration in the uterine epithelium and stroma. The expression of VEGF was thus evaluated at the transcriptional and translational levels. Semi-quantitative RT-PCR revealed a 2-fold reduction of VEGF transcripts and protein in the arsenic-treated rat uterus (Figure 6), indicating that arsenic not only down regulates the estrogen receptor, but also disrupts the downstream gene expression in the rat uterus.

### Effect of arsenic on the cell cycle regulatory proteins

**Figure 7:**
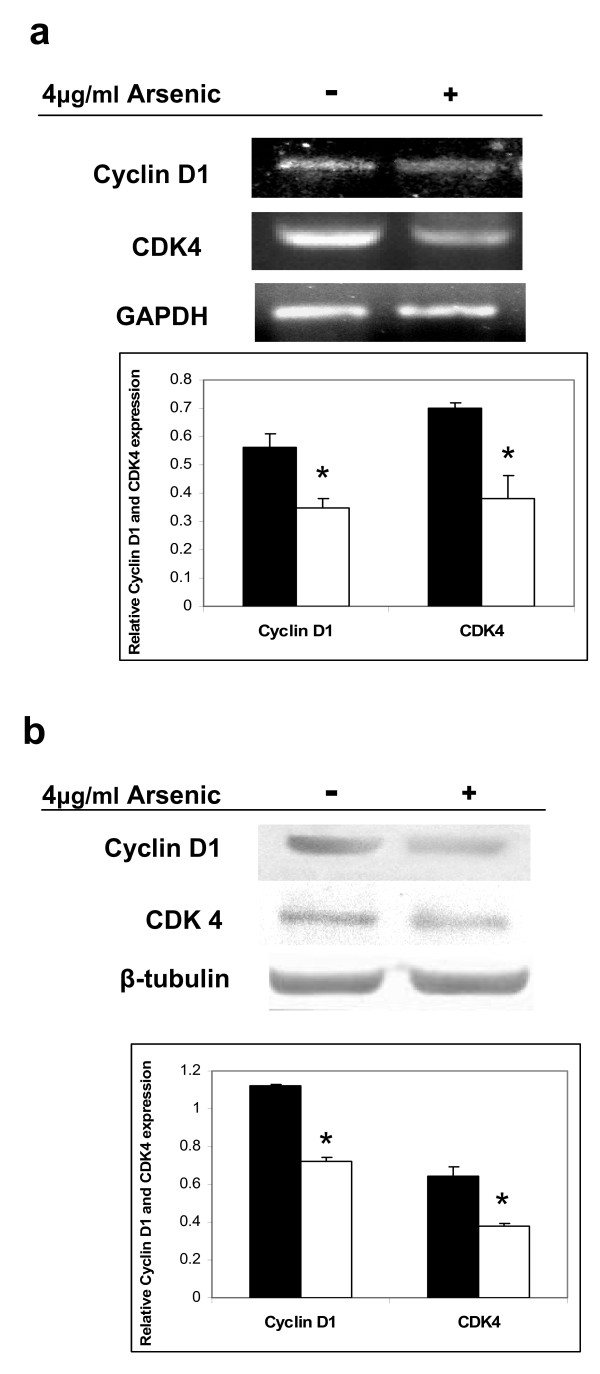
**Regulation of G1 cell cycle proteins by arsenic in rats**. Female rats were treated with arsenic at a dose of 4 μg/ml for 28 days. Cyclin D1 and CDK4 expressions were analyzed both at the mRNA levels (a) and protein levels (b), using GAPDH and β-tubulin as loading controls, respectively. It was observed that arsenic downregulated both cyclin D1 and CDK4 (white bars) in the uterus of Sprague-Dawley rats, as compared to the control animals (black bars). The data shown is a representative of the experiments carried out in triplicate.

It is well established that decreased levels of estrogen can affect the expression of the cell cycle regulators, especially those that are involved in the G1-S transition, chiefly cyclin D1 and CDK4. Concomitantly, we investigated if arsenic, which decreased the serum estradiol levels and estrogen receptor expression in female rats, would additionally alter the expression of cyclin D1 and CDK4. The results indicated significant reduction in cyclin D1 and CDK4 mRNA levels in uteri of rats exposed to arsenic, as compared to unexposed rats (Figure 7a). Concomitant downregulation in the expression of cyclin D1 and CDK4 proteins was also observed in uteri of rats exposed to arsenic treatment (Figure 7b).

## Discussion

In this study we report the effects of inorganic arsenic on the estrogen signaling pathway in rats, along with concomitant alterations of the uterine morphology and proliferation, to unravel the putative mechanisms behind reproductive failures associated with arsenic exposure. Rats were chronically (28 days) exposed to arsenite (4 μg/ml) in drinking water in order to establish a correlation between uterine pathology, serum estradiol and gonadotropin concentrations, and alterations in expression of the estrogen receptor and downstream components, involved with uterine tissue architecture and function, following arsenic treatment. It is evident from the results that arsenic exposure diminishes the circulating levels of both gonadotropins and estradiol. This is further supported by the fact that ovarian steroidogenesis involves enzymes regulated by the gonadotropins FSH and LH, both of which have been reported to be inhibited following exposure to arsenic [49]. Consequently, low serum levels of FSH and LH in arsenic-treated rats lead to reduction in estradiol production and thus secretion into circulation. Some reports have postulated that the decrease in gonadotropin levels may be due to an increase in plasma glucocorticoids in arsenic-treated rats, since increase in ACTH levels is known to suppress gonadotropin secretion [38].

The finding that arsenic exposure did not affect the total body weight during the exposure period, but decreased the uterine wet weight by almost 30% may be attributed to the fact that the effect of arsenic is very specific to the uterus in the tested time period. This is further supported by the fact that estradiol regulates uterine weight, and thus, low levels of estradiol lead to the specific reduction of uterine wet weight in the exposed animals. In addition, constant diestrus in arsenic-treated rats after 20 ± 2 days may also be due to low serum levels of estradiol [50-52].

In addition to altering the uterine wet weight, arsenic also leads to tissue degeneration in the uterus. The degenerative changes may be attributed to adverse effects caused by decreased serum estradiol levels, since uterine growth is primarily dependant on estradiol. The insufficient concentration of estradiol failed to maintain the normal uterine architecture and led to degeneration of the luminal epithelial cells and endometrial glands. Arsenic is known to generate reactive oxygen species and leads to oxidative damage to several components of the cell, including denaturation of proteins critical to cell functions [53-58]. Thus, degeneration of the uterine endometrial components may be associated not only with downregulated serum estradiol levels but also with increased production of reactive oxygen species following exposure to arsenic [56].

Arsenic is known to act as a potent environmental estrogen [32]. Subsequently, it was hypothesized that arsenic may mimic an estrogenic mechanism to induce lesions in the rat uterus, disrupt the estrogen signaling pathway and consequently lead to reproductive failures. Accordingly, the effect of arsenic on the expression of estrogen receptor, the estrogen responsive gene VEGF, and cell cycle regulatory proteins like CDK4 and cyclin D1 was investigated in the rat uterus. It is known that the estrogen receptor is a hormone-activated transcription factor which mediates the biological effects of estrogen in the target tissue by stimulating the expression of estrogen-regulated genes. The sensitivity of a given tissue to estrogen thus varies with the level of estrogen receptors present in it [59]. Our studies have revealed that arsenic treatment significantly downregulated the expression of ERα and its downstream element VEGF in the uterus, indicating that arsenic either suppresses the bonafide action of estradiol on the uterus by decreasing the expression of specific receptors, at both the mRNA transcript and protein levels, or acts via a parallel mechanism in the rat uterus that eventually disrupts the estrogen signaling pathway and the G1 cell cycle proteins responsible for cell proliferation. Estradiol-regulated VEGF is chiefly responsible for modulating in vivo angiogenesis in the uterus, and its downregulation by arsenic may be a primary cause for spontaneous abortions, still-births and other reproductive failures. The D-type cyclins are known to be rate-limiting for the progression through the G1 phase of the cell cycle [60]. In fact, a strong correlation between the expression of increased levels of cyclin D1 mRNA and ER over-expression has been reported [61]. In addition, estrogens are known to increase cell proliferation by recruiting resting cells into the cell cycle, reducing the length of G1 phase and promoting entry of cells to the S phase [62]. Arsenic, on the other hand, decreased the expression of uterine estrogen receptors, and consequently suppressed cell cycle progression and reduced the proliferation-promoting effects of estradiol in the rat uterus.

## Conclusions

It may thus be concluded that sodium arsenite is a nonsteroidal environmental estrogen that is responsible for reducing the serum levels of gonadotropins and estradiol, which in turn lead to uterine tissue degeneration and disruption of the estrogen signaling pathway. The effects of arsenic on the uterus may occur by reducing the expression of estrogen receptors and estrogen responsive genes, and/or by generating reactive oxygen species that lead to oxidative damage of the proteins involved in the estrogen signaling pathway, that regulate the uterine structure and function. Interestingly, liver toxicity assays carried out in our laboratory did not indicate significant differences in the SGPT or SGOT levels in the experimental animals as compared to the control ones (data not shown). Hence, it may be imperative to state that level of arsenic which failed to reduce general body weight of the rats or even affect the liver toxicity enzymes, were capable of bringing about such severe detrimental changes in the uterine physiology and steroid signaling pathway. Finally, our study demonstrates that arsenic at low but chronic doses, relevant to the exposure level in different parts of the world, is a major endocrine disruptor and thus, may be responsible for the different reproductive failures seen in women exposed to such levels of arsenic.

## List of abbreviations

DEPC: Diethyl Pyro Carbonate; EDTA: Ethylene Diamine Tetra Acetic Acid; FSH: Follicle Stimulating Hormone; GAPDH: Glyceraldehyde 3-Phosphate Dehydrogenase; HRP: Horse Radish Peroxidase; LH: Luteinizing Hormone; MuLV: Murine Leukemia Virus; NADPH: Nicotinamide Adenine Diphosphate Nucleotide; PBS: Phosphate Buffered Saline; PBST: Phosphate Buffer Saline Tween-20; RH: Relative Humidity; RIPA: Radio Immuno Precipitation Assay; RT-PCR: Reverse Transcriptase Polymerase Chain Reaction; VEGF: Vascular Endothelial Growth Factor

## Competing interests

The authors declare that they have no competing interests.

## Authors' contributions

AC carried out the treatment of animals and performed all the experiments. UC conceived the study, participated in its design, coordination, interpretation and analysis of the data. AC and UC drafted the manuscript. All authors read and approved the final manuscript.

## Authors' information

U.C. - Ph.D., Associate Professor, Department of Zoology, University of Calcutta

A.C. - M.Sc., Senior Research Fellow, Department of Zoology, University of Calcutta
